# The G428A Nonsense Mutation in FUT2 Provides Strong but Not Absolute Protection against Symptomatic GII.4 Norovirus Infection

**DOI:** 10.1371/journal.pone.0005593

**Published:** 2009-05-18

**Authors:** Beatrice Carlsson, Elin Kindberg, Javier Buesa, Gustaf E. Rydell, Marta Fos Lidón, Rebeca Montava, Reem Abu Mallouh, Ammi Grahn, Jesús Rodríguez-Díaz, Juan Bellido, Alberto Arnedo, Göran Larson, Lennart Svensson

**Affiliations:** 1 Division of Molecular Virology, University of Linköping, Linköping, Sweden; 2 Department of Forensic Genetics and Forensic Toxicology, National Board of Forensic Medicine, Linköping, Sweden; 3 Department of Microbiology, School of Medicine and Hospital Clínico Universitario, University of Valencia, Valencia, Spain; 4 Department of Clinical Chemistry and Transfusion Medicine, Sahlgrenska University Hospital, Göteborg, Sweden; 5 Sección de Epidemiología, Centro de Salud Pública, and CIBER-ESP, Castellón, Spain; Health Protection Agency, United Kingdom

## Abstract

In November 2004, 116 individuals in an elderly nursing home in El Grao de Castellón, Spain were symptomatically infected with genogroup II.4 (GII.4) norovirus. The global attack rate was 54.2%. Genotyping of 34 symptomatic individuals regarding the *FUT2* gene revealed that one patient was, surprisingly, a non-secretor, hence indicating secretor-independent infection. Lewis genotyping revealed that Lewis-positive and negative individuals were susceptible to symptomatic norovirus infection indicating that Lewis status did not predict susceptibility. Saliva based ELISA assays were used to determine binding of the outbreak virus to saliva samples. Saliva from a secretor-negative individual bound the authentic outbreak GII.4 Valencia/2004/Es virus, but did not in contrast to secretor-positive saliva bind VLP of other strains including the GII.4 Dijon strain. Amino acid comparison of antigenic A and B sites located on the external loops of the P2 domain revealed distinct differences between the Valencia/2004/Es and Dijon strains. All three aa in each antigenic site as well as 10/11 recently identified evolutionary hot spots, were unique in the Valencia/2004/Es strain compared to the Dijon strain. To the best of our knowledge, this is the first example of symptomatic GII.4 norovirus infection of a Le^a+b−^ individual homozygous for the G428A nonsense mutation in *FUT2*. Taken together, our study provides new insights into the host genetic susceptibility to norovirus infections and evolution of the globally dominating GII.4 viruses.

## Introduction

Noroviruses (NoV) have emerged as an important cause of gastroenteritis outbreaks in institutions such as elderly nursing homes, hotels, hospitals and schools [1], [2]. NoV contains a linear positive-sense single stranded RNA genome of ∼7.7 kb in length, surrounded by a 530 amino acid (aa) long capsid protein (Norwalk strain), which is folded into two major domains; the conserved S (shell) domain and a more variable P (protruding) domain [3]. Recent studies have suggested that the protruding NoV capsid domain, subdivided into P1-1, P1-2 and P2, bear antigenic determinants affecting the immunological response and host specificity [4]–[8].

Transmission of NoV occurs predominantly through contaminated food, water, fomites, and by person-to-person through the fecal-oral route [9]. Evidence of protective immunity to NoV is controversial; short-term to no immunity has been reported [10]–[12] and antibodies do not seem to provide protection, at least not against the genogroup I Norwalk virus [13]. Furthermore, volunteer studies have shown that a subset of individuals remain uninfected even after repeated challenges [10], [12], [14]. This information, together with the fact that only low infectious doses are required for infection, and that attack rates seldom exceeds 70% [15], suggest that some type of inherited factors act to prevent certain individuals from symptomatic NoV infection. Recent studies have shown that secretor status; the ability to express histo-blood group antigen (HBGA) on mucosa and in secretions may affect the risk of being symptomatically infected by NoV [16]–[22].

Non-secretors (sese), who do not express the Fuc-TII α1,2-fucosyltransferase and consequently do not express H type 1 or Lewis b (Le^b^) antigens, have been shown to be less susceptible or even resistant to authentic NoV infections [16], [19], [21], [22]. Approximately 20% of Northern Europeans and Caucasian Americans are secretor-negative [23]. Furthermore, sero-epidemiology studies have shown that secretors have significantly higher antibody titers and prevalence against NoV than non-secretors [24]. However, the fact that certain non-secretors are NoV antibody-positive, suggests that secretor-independent infections do occur [25], [26], maybe with distinct virus strains.

Several mutations are known in the *FUT2* gene [23], and some of them show high ethnic specificity [27], [28]. The G428A nonsense mutation is typically found in the Caucasian population [23], [27] whereas the nonsense C571T mutation is found mainly in Pacific Islanders [29]. Both these mutations give rise to an early stop codon, giving a truncated non-functional protein. Homozygous carriers of any nonsense mutation in the *FUT2* gene are called non-secretors. Homozygous carriers of a missense mutation at position 385 (A>T) are so called “weak secretors”, expressing lower levels of ABH antigen in saliva and, if Lewis positive, a Lewis (a+b+) phenotype on erythrocytes [30].

In vitro binding studies have suggested that not only secretor status but also Lewis status may affect susceptibility to NoV [31], [32]. However, different strains show different binding patterns, with the worldwide dominating genogroup II.4 strains expressing the broadest histo-blood group-binding pattern and thought to be able to infect secretor-positive individuals of all ABO blood group types irrespective of Le status [31].

Previously, only secretor-positive individuals have been symptomatically infected with the globally dominating GII.4 virus [16], [19], [21]. However, Lindesmith and co-workers have shown that a GII.4 strain detected in 2002 (2002a), bound not only secretor-positive but also to secretor-negative saliva under certain conditions [33], hence indicating infection with GII.4 virus also in non-secretors. In this study we report for the first time of symptomatic GII.4 NoV infection of an individual homozygous for the G428A nonsense mutation, a mutation that previously has been shown to provide complete protection from authentic GII.4 NoV disease [19], [21]. Furthermore, we show that antigenic regions A and B in the P2 domain as well as 10/11 recently identified evolutionary hot spots proposed to be associated with molecular evolution [34] are distinct in the outbreak virus.

## Results

### Description of the outbreak

During November 6^th^ to November 20^th^ 2004 an outbreak of acute gastroenteritis consisting of 116 cases occurred in an elderly nursing home in El Grao de Castellón, Spain. The facility consists of a building exclusively dedicated to this purpose and includes 65 double-rooms in two floors occupied by 130 residents. In addition, 30 old persons visited the residence daily, which serves as a day-care center. The nursing home employs 90 healthcare workers and other staff members, 58 of whom were interviewed.

Out of the 130 residents in the facility, 75 (57.7%) persons suffered acute gastroenteritis. Sixteen (61.5%) of 26 out-patients were interviewed and 25 (43.1%) of the staff members experienced an episode of acute gastroenteritis, with a total of 116 persons affected.

The first three cases were reported on the 6^th^ of November 2004 and since then other residents developed symptoms of acute gastroenteritis with progression towards the peak of the outbreak on November 12^th^ 2004, with 44 cases on that date. The global attack rate was 54.2%. The most common symptoms were diarrhea (79%) and vomiting (66%), with fever (>37.5°C) recorded in 13% of the patients. The average duration of symptoms was less than two days. Five patients were hospitalized, but no casualties were observed.

### The outbreak was caused by a GII.4 strain

**Figure 1:**
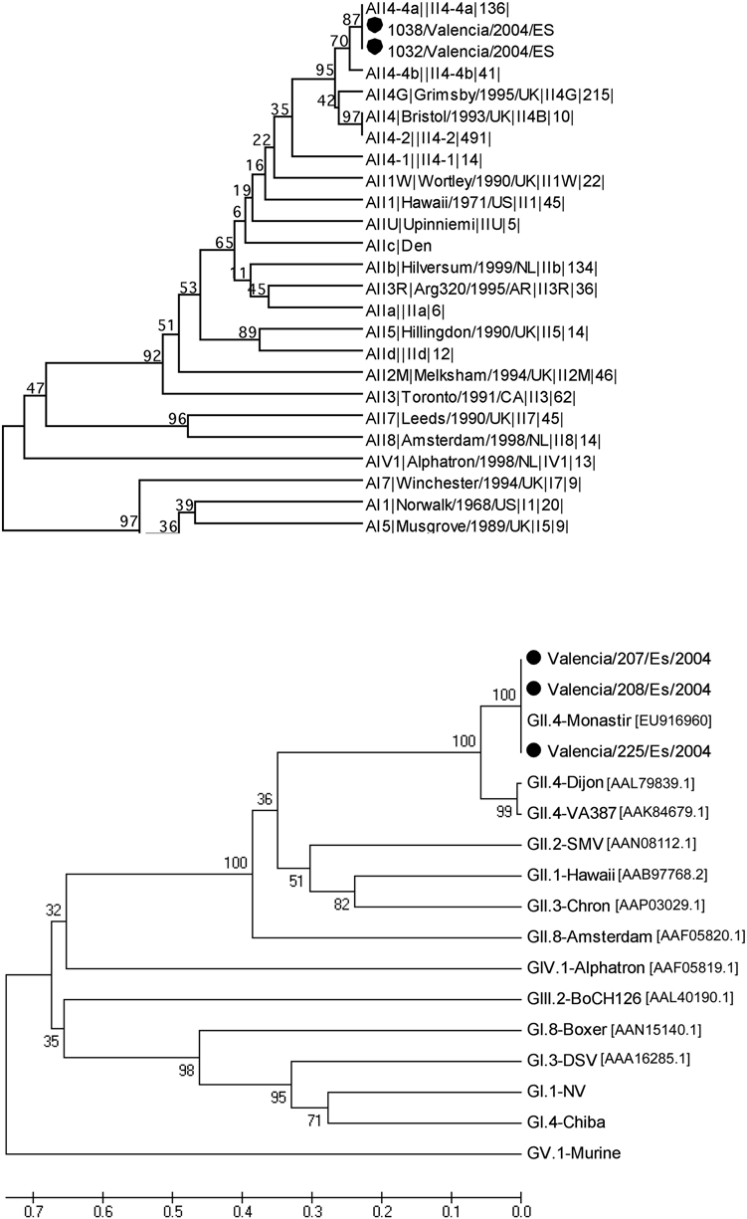
Phylogenetic analysis of the outbreak Valencia/2004/Es strain. A) Phylogenetic tree of the NoV RNA polymerase gene (region A in ORF1) from the outbreak (Valencia/2004/Es) and reference strains, obtained from the European Food-borne viruses database [2]. The phylogenetic tree was constructed using the UPGMA clustering method with distance calculation using the Jukes-Cantor correction for evolutionary rate by Molecular Evolutionaty Genetics Analysis (MEGA version 2.1). B) Phylogenetic tree of the outbreak (Valencia/2004/Es) capsid P2 domain (aa 279 to 405) and selected reference strains. The phylogenetic tree was constructed using the UPGMA clustering method with distance calculation using the Poisson correction for evolutionary rate by Molecular Evolutionaty Genetics Analysis (MEGA version 4.1).

NoV were detected by RT-PCR in 27 out of 33 (81.8%) fecal samples tested from symptomatic patients, both residents and healthcare workers. As no other enteric virus (rotavirus and enteric adenovirus) or bacteria were detected from the patients it was concluded that NoV was the etiological agent of this outbreak. Sequencing of a portion of the RNA polymerase gene as well as the P2 region of the capsid gene, from four and three different specimens respectively, confirmed that the outbreak was caused by GII.4a-2004 variant virus (Figure 1A and 1B).

### The G428A nonsense mutation in *FUT2* provides strong but not absolute protection against symptomatic GII.4 NoV infection

To investigate any association between mutation in the *FUT2* gene and resistance to symptomatic infection, genotyping was performed to identify individuals as secretor-negative, heterozygous secretors or homozygous secretors (Table 1). None of the individuals were carriers of the mutations at nt 385 (weak secretor) or at nt 571 in the *FUT2* gene. Of the symptomatic individuals 38.2% (13/34) were homozygous secretors and 58.8% (20/34) were heterozygous secretors. Most interesting was that one (2.9%) symptomatic female patient (patient A) was found to be homozygous carrier of the G428A mutation and hence a non-secretor. Among the asymptomatic/non-exposed individuals, 15.4% (4/26) were homozygous secretors, 23.1% (6/26) were heterozygous and 61.5% (16/26) were non-secretors. Thus significant difference (P<0.001) in susceptibility to symptomatic NoV infection was found between secretors and non-secretors.

**Table 1:** Strong but not absolute correlation between the G428A *FUT2* nonsense mutation and symptomatic NoV infection.

	No (%) of patients	SeSe	Sese	sese	LeLe/Lele sese (Le^a+b−^)	LeLe/Lele SeSe/Sese (Le^a−b+^)	lele SeSe/Sese/sese (Le^a−b−^)
**Symptomatic**	34 (56.7)	13* (38.2)	20* (58.8)	1* (2.9)	1# (3.8)	23# (88.5)	2 (7.7)
**Asymptomatic/Nonexposed**	26 (43.3)	4* (15.4)	6* (23.1)	16* (61.5)	9# (50)	5# (27.8)	4 (22.2)
**Total**	60	17 (28.3)	26 (43.3)	17 (28.3)	10 (22.7)	28 (63.6)	6 (13.6)

* SeSe and Sese^428^ vs. se^428^se^428^ P<0.001.

# Le^a+b−^ vs. Le^a−b+^ P<0.001.

The Lewis-genotype could be determined in 44 of 60 individuals.

SeSe and LeLe: homozygous wildtype for *FUT2* and *FUT3*.

Sese and Lele: heterozygous for the inactivating mutations of *FUT2* and *FUT3*.

sese and lele: homozygous for the inactivating mutations of *FUT2* and *FUT3*.

Le^a+b−^: Secretor-negative Lewis-positive phenotype.

Le^a−b+^: Secretor-positive Lewis-positive phenotype.

Le^a−b−^: Lewis-negative phenotype.

### Lewis status was not identified as a susceptibility marker for symptomatic NoV GII.4 infection

While Le^a−b+^ but not Le^a+b−^ individuals are highly susceptible for symptomatic NoV infections, little information is available regarding Le^a−b−^ individuals in authentic NoV outbreaks. To determine whether Lewis status affected the susceptibility of infection, *FUT3* genotyping was performed using PCR with sequence specific primers (PCR-SSP) [35]. Forty four of 60 individuals (73.3%) were genotyped and the results showed that six individuals were Lewis-negative due to being homozygous carriers of inactivating mutations at nt 202 and 314 (two individuals), at nt 59 and 1067 (one individual) and the remaining three being compound heterozygous at nt 202, 314, 59, 508 or 1067. Two Le^a−b−^ individuals were secretor-positive and four were non-secretors (Table 1). The two secretor-positive Lewis-negative individuals were both symptomatically infected, whereas none of the four Lewis-negative non-secretors became ill.

### Saliva from secretors and a non-secretor as well as Lewis-positive and Lewis- negative individuals bound the outbreak virus strain

**Figure 2:**
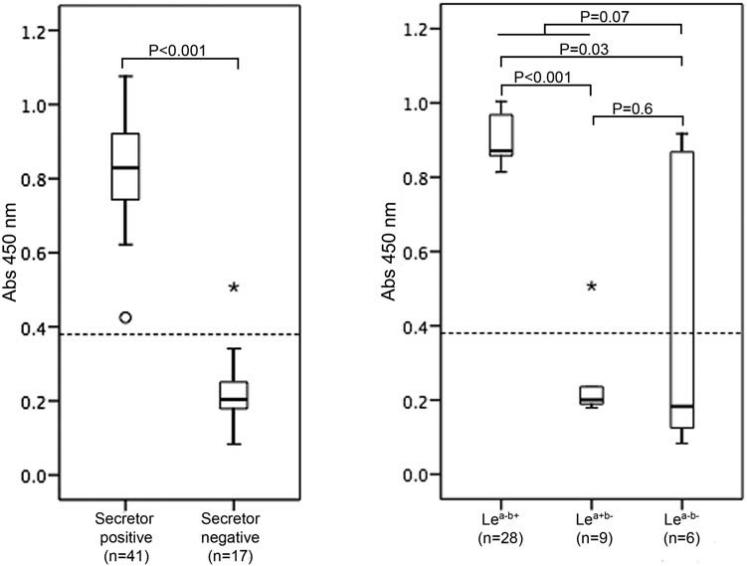
Binding of authentic Valencia/2004/Es virus to saliva. Binding of saliva from secretors and non-secretors to the GII.4 outbreak virus. Dotted line represents cut-off value. Cut-off value (0.380) was three times the mean OD_450_ of three known negative control samples. The box shows interquartile range; the range between the first and third quartiles. Median is marked as a horizontal line within the box. Whiskers represent samples not more than 1.5 times the box width away from the box. The ring represents a sample within 1.5–3 box lengths from upper or lower edge of the box and the asterisk marks an extreme case (a value more than 3 box (lenghts from the edge of the box), here representing patient B (asymptomatic non-secretor).

Several previous studies have shown that saliva of secretor-positive but not of secretor-negative individuals can bind NoV VLP [17] and authentic virus [19]. To investigate the property of our outbreak virus, the virus was incubated with saliva from symptomatic and asymptomatic/non-exposed individuals in an ELISA assay. Figure 2 shows that saliva from secretors bound the outbreak virus, but interestingly also saliva from one asymptomatic non-secretor (Le^a+b−^), referred to as patient B. Because of the restricted amount of saliva (at the time for first sample collection), patient A (symptomatically infected non-secretor, Le^a+b−^) could not be tested. Figure 2 shows that except for the binding of patient B, saliva of Le^a−b+^ but not of Le^a+b−^ individuals bound the virus (P<0.001) but more interestingly that Lewis status (positive vs negative) could not predict binding. The virus bound to saliva from two of six Le^a−b−^ individuals both of which were secretors. The four Le^a−b−^ individuals whose saliva could not bind the virus were all non-secretors.

**Figure 3:**
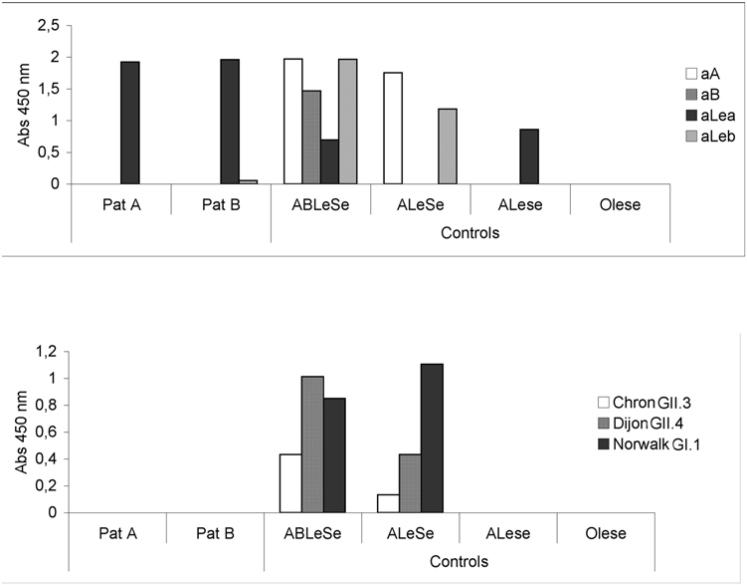
Detection of histo-blood group antigens and VLP binding to saliva from patient A and B. A) ABO and Lewis histo-blood group phenotyping of saliva samples from the non-secretor patients A and B confirms a Le^a+b−^ phenotype. Saliva samples were coated in microtiter wells and incubated with aA (anti A), aB, aLe^a^ or aLe^b^ antibodies separately. The limit of detection for each antibody has been subtracted from each bar. Both patients reacted strongly only with the aLe^a^ antibody and not with the aLe^b^, aA or aB antibodies and were thereby classified as Le^a+b−^. A, AB or O denotes blood groups, Le or le denotes Lewis positive or Lewis negative, respectively, and Se or se denotes secretors or non-secretors. B) Saliva from non-secretor patients A and B or non-secretor controls do not bind Chron, Dijon or Norwalk VLPs whereas saliva from secretors bind all three strains.

To confirm the histo-blood group phenotype of the saliva samples from the two non-secretors, ABO and Lewis phenenotyping was performed on these saliva samples. Consistent with the *FUT3* Lewis-positive genotyping, the two saliva samples from the secretor-negative individuals (patient A and B) were identified as Le^a+b−^ by phenotyping (Figure 3A). Furthermore, neither patient A nor B expressed A or B antigen in saliva, which is, indirectly, further support that they indeed were non-secretors.

To determine if saliva (collected at a second time point) of patient A (symptomatically infected non-secretor) and patient B (asymptomatic non-secretor whose saliva bound the outbreak virus) would bind NoV from different genogroups and genotypes, a saliva VLP assay was established. Figure 3B shows that saliva from patients A and B, in contrast to secretor-positive controls (ABLeSe and ALeSe individuals) did not bind VLP from GI.I (Norwalk strain), GII.3 (Chron1 strain) or GII.4 (Dijon strain), the latter belonging to the same genotype as the outbreak strain. Figure 3B also shows that saliva of secretor-negative controls (ALese and Olese individuals) did not bind any of the VLPs.

### The outbreak Valencia strain have distinct amino acids in antigenic A and B regions of the P2 domain compared to the Dijon strain

The fact that saliva from a non-secretor (patient B) recognized the outbreak virus strain and one non-secretor become ill (patient A) raised the question if the virus had an unique aa sequence in the P2 domain of the capsid protein. To address this question the P2 domain (nt 667 to nt 1382) of three random samples (no 207, 208, 225) was sequenced and a BLAST search on the NCBI server was performed. This revealed not only that the Valencian isolates were identical in the P2 domain but also that the outbreak strain was most similar to the GII.4 strain Monastir/2003/Tun [EU916960] isolated in Tunisia 2003 [36]. The Valencia/2004/Es and the Monastir/2003/Tun strains were identical in every position (aa 248 to 420) except for a conserved substitution at residue 356, where a valine in the Monastir strain was changed for an isoleucine in the Valencia strain.

**Figure 4:**
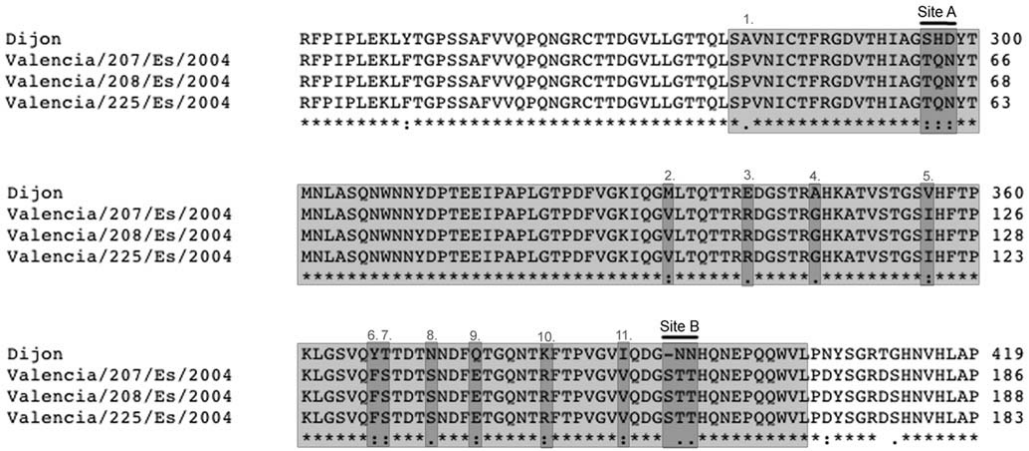
Comparison of antigenic sites between the outbreak Valencia/2004/Es and Dijon strain. The outbreak Valencia/2004/Es strain have distinct amino acids in antigenic A and B regions of the P2 domain compared to the Dijon strain. Amino acid alignment (Clustal W 1.8 with default parameters on the European Bioinformatics Institute server) of partial capsid protein (aa 241 to 419) of the Dijon171/96 [AF472623] and the outbreak Valencia/2004/Es strains. The Valencia/2004/Es sequences, are obtained from three randomly chosen patients (no 207, 208 and 225). The aa constituting the P2 domain (aa 279 to 405) are shaded in light grey. Antigenic site A (aa 296–298) and site B (393–395) proposed by Allen et al. [34] are indicated. The Valencia/2004/Es has a TQN and a STT motif in the A and B site, while the Dijon strain have a SHD motif in site A and a –NN motif in site B. Besides from the A and B site, 11 additional substitutions (marked number 1 to 11) were found when comparing the capsid P2 domain of Valencia and Dijon, 10 out of these 11 (number 2 to 11) correlated with evolutional hot spots (highlighted by dark grey) identified by Allen and coworkers [34].

Despite that saliva from patient B bound the outbreak virus, it did not bind the Dijon VLP used in the binding assay, even though both strains belong to GII.4. This observation suggested that the Valencia strain might have HBGA-receptor specific domains different from the Dijon strain. To investigate this, the aa of the P2 domain of Valencia and Dijon were aligned and compared (Figure 4). Particular interest was given to antigenic region A and B of the P2 domain that previously have been associated with molecular evolution [34]. As illustrated in Figure 4 antigenic region A and B of the Valencia strain are distinct from the Dijon strain, all 3 aa in each antigenic site are different. The Valencia strain have a TQN and a STT motif in the A and B site respectively, while the Dijon strain have a SHD motif in site A and a –NN motif in site B. Besides the A and B sites, 11 additional substitutions (marked number 1–11, Figure 4) were found when comparing the capsid P2 domain of Valencia and Dijon, 10 (number 2 to 11, figure 4) out of these 11 correlated with evolutional hot spots identified by Allen and coworkers [34].

## Discussion

In this study we describe an outbreak with a GII.4 NoV affecting both secretors and a non-secretor, but with significantly higher susceptibility among secretors (76.7%, 33/43) compared to non-secretors (5.9%, 1/17) (P<0.001). One female non-secretor (patient A) was symptomatically infected and virus was found in her stool. *FUT2* genotyping identified 38.2% (13/34) of the infected individuals as homozygous wild types, 58.8% (20/34) were heterozygous secretors and one (2.9%) was a non-secretor. Among asymptomatic/non-exposed individuals, 15.4% (4/26) were homozygous secretors, 23.1% (6/26) were heterozygous and 61.5% (16/26) were non-secretors. The total frequency of non-secretors was 28.3% (17/60) and was thus higher than the frequency of the Northern European population, which is approximately 20% [19], [21], [23], [37]. Saliva from a non-infected non-secretor (patient B) recognized the outbreak virus in a saliva-based ELISA, suggesting that the virus may have different binding properties from what is generally observed among GII.4 NoV strains. A similar finding has previously been reported by Lindesmith and co-workers [33] who demonstrated binding of a GII.4 virus to saliva from both secretors and non-secretors.

Most surprisingly, we found that saliva from the non-secretor did not only fail to recognize the Chron GII.3, Norwalk GI.1 but also the GII.4 Dijon VLP, even though both Valencia/2004/Es outbreak virus and the Dijon strains belong to GII.4. This observation implied that the Valencia strain might have HBGA-receptor specific domains different from the Dijon strain, suggesting that association of histo-blood group antigens with susceptibility to NoV infection may be strain-specific rather than genogroup dependent [38]. Indeed, recent data suggest variations in HBGA recognition within the GII.4 genotype [33].

To investigate the possibility that the outbreak virus had unique aa sequence and structural properties in the capsid protein, the P2 domain (aa 248 to 420), of 3 isolates (patients no 207, 208 and 225) were sequenced and BLAST search was performed. This revealed that the outbreak strain belonged to the globally dominating GII.4 genotype and was most similar to the Monastir/2003/Tun [EU916960] strain isolated in Tunisia 2003. The Valencia and the Monastir capsid were identical in the investigated region except for a conserved substitution at residue 356, where a valine in the Monastir strain was changed for an isoleucine in the Valencia strain. Cao and coworkers [6] have previously proposed from crystal structure studies that the residues involved in binding of the A and B trisaccharide to the GII.4 virus (VA387 strain) are located at the dimer interface and includes residues 343, 344, 345 and 374 from one protomer and 441, 442 and 443 in the other. A putative carbohydrate-binding patch consisting of residues 329, 373, 375 and 377 located in the P2 domain has also been suggested by Chakravarty and coworkers [39]. However, since the sequencing of the Valencia capsid P2 domain did not reveal any novel mutations in these regions, it is possible that the outbreak virus may contain other structural determinants that would alter receptor binding and antigenicity, and thus explaining infection of the secretor-negative individual and binding to secretor-negative saliva.

One of the driving forces for NoV evolution, particularly in the protruding part of the capsid protein, is probably immune evasion [4], [7], [33], [34], [40]. Recent studies have shown that a given NoV genotype predominates in a season, such as GII.4 in Europe and the United States [21], [41]–[44]. This predominance of a given genotype or cluster is followed by a sharp drop in prevalence of the genotype in the following season [40], [44], which again is followed by the emergence of a genetically distinct lineage. Thus, periods of phenotypic stasis are followed by the emergence of novel epidemic strains [33], [40].

Allen and co-workers [34] have found two antigenic regions (site A and B) in the P2 domain of the VA387 crystal structure, where aa substitutions in this area have impact on the biochemical properties as well as the entire structure of the P2 domain. These positions, located to external parts in the capsid, are part of exposed loops and thus changes in site A and B may have a strong association with the emergence of novel NoV strains [34]. Since the non-secretor saliva bound the outbreak strain but not the Dijon strain, we decided to compare the antigenic site A and B of the Valencia/2004/Es strain with the Dijon strain. Alignment of the P2 domain from Valencia/2004/ES and Dijon revealed that all three amino acids in both antigenic region A and B were unique in Valencia/2004/Es as compared to the Dijon strain, possibly causing the differences in binding properties between the virus strains.

The Valencia/2004/Es strain have a TQN and a STT motif in the A and B site respectively, while the Dijon strain have a SHD motif in site A and a –NN motif in site B. According to Allen and coworkers, the motif found in the Valencia strain characterize GII.4 strains isolated in 2004, 2005 and 2006, while the motif from the Dijon strain are found in strains circulating during 1997, 1998 and 1999 [34]. This pattern correlates well to the year of isolation both for the Valencia (2004) and the Dijon strains (1996). Besides from the A and B site, 11 additional substitutions were found when comparing the capsid P2 domain of Valencia/2004/ES and Dijon strains, 10 out of these 11 correlated with evolutional hot spots identified by Allen and coworkers [34]. We therefore speculate that the aa differences observed in site A, B and in the evolutional hot spots, may affect the structural and electrostatic properties of the capsid protein, perhaps giving a clue to the different binding patterns observed between the Valencia/2004/ES and the Dijon strain. However, when aligning the Dijon and the Valencia/2004/ES strains, we also observed differences in aa surrounding the A and B trisacharide binding site proposed by Cao et al [6]. These positions include e.g. residue 346, 372, 389 but also residue 393 (part of the antigenic site B identified by Allen and coworkers [34]) which all differ between the Dijon and the Valencia/2004/ES strains. Of these positions, 393/394 are also identified by Lindesmith and coworkers, as one site out of six in the NoV capsid operating under positive selection [33]. Lindesmith and coworkers suggest that the aa in position 393/394 may play an key role in receptor binding and impact immunogenic properties of the virus [33]. Also a study by Siebenga and coworkers describing epochal evolution of GII.4 capsid proteins, identified position 393/394 as a hypervariable site during evolution [40]. We thereby can not role out that also the observed differences surrounding the A and B trisacharide biding site, perhaps in combination with the changes in antigentic site A and B may contribute to the unusual saliva binding properties observed in the outbreak virus. Unfortunately, no host genetic susceptibility data are available for the Monastir/2003/Tun strain. This would have been most informative since the capsid protein of the Monastir/2003/Tun are most similar to the Valencia/2004/ES strain and contain identical aa in both antigenic site A and B.

Another possibility that might explain the uncommon saliva binding property observed for the Valencia/2004/Es strain could be the unexpected appearance of type 1 chain ABH or Lewis b antigens in patient A and B. To investigate this, Lewis genotyping and AB(O) and Lewis phenotyping was performed on saliva from these two patients. These investigations concluded that the patients are indeed Le^a+b−^ non-secretors. However, additional structures used by NoV for binding, may be present in the saliva of these individuals.

Although sero-epidemiology studies have shown that secretors have significantly higher antibody titers and prevalence against NoV than non-secretors [24], the fact that certain non-secretors are NoV antibody–positive [24], [26], suggests that secretor-independent infections do occur. Also, a few documented NoV infections of non-secretors have been reported [25], [26].

Six of 44 investigated individuals were found to be Le^a−b−^ by genotyping. Four of these individuals were secretor-negative and two were secretor-positive. Of these, only the secretor-positive Le^a−b−^ individuals were symptomatically infected. We therefore speculate that secretor status but not Lewis status may correlate with NoV susceptibility. Further support for this hypothesis is also the observation that Lewis status did not predict binding of virus to saliva (Figure 2). The conclusions are consistent with a previous observation by Larsson and co-workers [24] who found that antibody titers and prevalence to NoV correlate with secretor rather than with Lewis status. This is also in agreement with the result from Bucardo and co-workers who found NoV susceptibility to be independent of Lewis-phenotype [45].

While the outbreak-virus infected as well as recognized saliva from a non-secretor, current information cannot explain why not all non-secretors were infected or why the virus did not recognize all non-secretor saliva. Possible explanation for this might be that the infected secretor-negative individual may express receptors present in only a limited number of non-secretors, or an unusual high concentration of a receptor commonly found at low concentration in non-secretors. Rydell and co-workers recently showed that GII.3 and GII.4 VLPs could bind Sialyl-Lewis x on neoglycoproteins, suggesting that this may be a possible determinator of NoV tropism [46]. Also, Taube and co-workers have suggested a role of sialic acid moieties in murine NoV attachment to murine macrophages [47]. Furthermore, Tamura and co-workers have shown that NoV GII VLPs efficiently bind surface heparin sulfate on the surface of different cell lines [48]. Together these studies indicate a role of sialylated structures or heparin sulfate in NoV cell tropism. Another possibility is that certain individuals are immune. This speculation is supported with data from other studies showing that not all secretors are infected in a given outbreak [19]. Furthermore, short term immunity have been demonstrated in volunteer studies [12].

In conclusion we show for the first time symptomatic NoV infection caused by a GII.4 strain in a secretor-negative Le^a+b−^ individual homozygously mutated at nt 428 of *FUT2*.

## Materials and Methods

### Subjects and samples

Fecal samples, collected from 26 symptomatic residents and from 7 staff members, were sent to the local hospital laboratory for bacteriology and virology investigation. Enteropathogenic bacteria (*Salmonella*, *Shigella*, *Campylobacter*, *Yersinia* and *Aeromonas* species) were investigated by conventional bacterial culture procedures [49] and rotavirus and adenovirus were analyzed by enzyme immunoassay (Premier Rotaclone and Premier Adenoclone 40/41, Meridian Bioscience Inc., Cincinnati, Oh.) and NoV by RT-PCR. Saliva samples were collected from 39 residents, including symptomatic patients and asymptomatic controls and from 21 health care workers of which 11 got sick and 10 remained asymptomatic (controls).

### Characterization of the nonsense *FUT2* mutations G428A, C571T and the missense mutation A385T

The secretor genotype was determined by pyrosequencing as described [37]. Briefly, DNA was extracted from 200 µl of saliva using QIAamp® DNA Mini kit (Qiagen, Hilden, Germany). Extracted DNA was stored in TE-buffer in Eppendorf tubes at −20°C until PCR amplification. For PCR amplification forward primers 5′-BIOTIN-GAT GGA GGA GGA ATA CCG CCA C-3′ (FUT2 428), 5′CGA CTG GAT GGA GGA GGA ATA C-3 (385) and 5′-BIOTIN-GCA CCT TTG TAG GGG TCC A-3′ (571) were used together with reverse primers 5′-TGG GCC TCC TCC CGC ACG T-3′ (428), 5′-CGG TGA AGC GGA CGT ACT-BIOTIN-3′ (385) and 5′-CTT CCA CAC TTT TGG CAT GAC-3′ (571). For sequencing the following primers were used: 5′-GGT GGT GGT AGA AGG TC-3′ (FUT2 428), 5′-GAG GAA TAC CGC CAC-3′ (385) and 5′TGG ACA TAG TCC CCT C-3′ (571).

FUT2 genotyping for mutations G428A, C571T and A385T was independently also performed by PCR-SSP as published [50].

### Determination of Lewis genotype and Lewis and AB phenotypes of saliva

Lewis genotype was determined using PCR-SSP as described elsewhere [50], detecting the mutations T59G, T202C, C314T, G508A and T1067A of the FUT3 gene. Lewis phenotypes were identified in an ELISA assay [46] using flat-bottom MaxiSorp Microtiter plates (NUNC, Roskilde, Denmark), anti blood group A (ABO1 clone 9113D10) and anti B (ABO2 clone 9621A8) antibodies (Diagast, Loos Cedex, France), anti Le^a^ (Seraclone, LE1 clone 78FR 2.3) and anti Le^b^ (Seraclone LE2 clones LM129-181 and 96 FR2.10) antibodies (Biotest AG, Dreieich, Germany) and finally HRP conjugated goat anti-mouse IgG (170-6516, Bio-Rad, Hercules, CA, USA.) and TMB (T0440, Sigma) as substrate. The absorbance values were read at 450 nm (Labsystems iEMS Reader MF, Labsystems, Helsinki, Finland) and average values for duplicate wells calculated.

### Binding of the outbreak NoV to saliva

To investigate if the outbreak strain could bind to saliva from symptomatic and asymptomatic individuals, a saliva-based ELISA was performed as described [19], [51] with some modifications. Saliva samples were boiled, centrifuged at 10,000×g for 5 min, and diluted 1∶500 in coating buffer (0.1 M carbonate-bicarbonate buffer, pH 9.6). After 2 h of incubation at 37°C followed by overnight incubation at 4°C, the plates were washed three times with washing buffer (0.9% NaCl 0.05% Tween 20) and blocked (3% bovine serum albumin, BSA in PBS) for 60 min at 37°C. A NoV stool suspension in PBS (10% w/v) was diluted 1∶2 in PBS with 0.05% Tween 20 and 0.5% BSA, and incubated for 2 h at 37°C followed by washing of the plates three times with washing buffer (0.9% NaCl 0.05% Tween 20) and then incubation with peroxidase-labeled genogroup I and II-specific NoV polyclonal antibody (DAKO, Denmark) for 1 h at 37°C. The reaction was developed using TMB (ICN Biochemicals) and the plate was read at 450 nm. The cut-off value was three times the mean OD_450_ value of three known negative control samples.

### Binding of NoV VLP to saliva

Norwalk (GI.1) and Dijon (GII.4) purified VLP was a kind gift from Jacques le Pendu and were used essentially as described above. Briefly VLP (0.16–2 µg/ml depending on VLP) was diluted in dilution buffer (0.5% BSA and 0.05% Tween 20 (Sigma) in PBS) and added to each saliva-coated well, and incubated at 37°C for 1.5 h. Following 3× washes appropriate anti NoV antisera was incubated for 1.5 h at 37°C. After washing HRP conjugate was added and incubated for 1.5 hr at 37°C followed by 3× washes and development by TMB as described above. The Chron1 (GII.3) VLP is a construct from a NoV strain cloned from an patient with a chronic NoV infection [4]. Production of recombinant VLP was done in Sf9 cells. Briefly Sf9 cells were infected and harvested 5 days p.i. Cells and media were then centrifuged at 2000 rpm for 10 min and the supernatant collected and pelleted by centrifugation at 30 000 rpm, 2 h in SW41Ti and resuspended in PBS followed by purification in a sucrose gradient. Purity and integrity was determined by Coomassie staining and western blot [46].

### RNA extraction and RT-PCR

Fecal suspensions (10% w/v in PBS) were clarified by low speed centrifugation. RNA was extracted by using the QIAmp viral RNA kit, according to the manufacturer's instructions (QIAGEN, Hilden, Germany). Purified RNA was then resuspended in 50 µl of RNase-free water and used as template for RT-PCR using primer pairs JV12/JV13 [52].

### Virus genotyping

Genotyping was performed by nucleotide sequencing of the PCR products obtained with primers JV12/JV13 (viral RNA polymerase gene) and Mon381/Mon383 (viral capsid gene) [53]. Sequencing was carried out in both directions using the BigDye Terminator cycle sequencing kit (Perkin-Elmer) on an automated ABI PRISM model 377 machine (Applied Biosystems). Sequence alignments were carried out by using ClustalW1.8 with reference strains obtained from the Foodborne Virus in Europe (FBVE) database (https://hypocrates.rivm.nl/bnwww/Divine-Event/index.html). A dendrogram was constructed using the UPGMA clustering method with distance calculation using the Jukes-Cantor correction for evolutionary rate by Molecular Evolutionaty Genetics Analysis (MEGA version 2.1).

### PCR amplification of NoV capsid P2-region

For the reverse transcriptase reaction 5 µl (0.5 µg) pd(N)_6_ primer (Amersham Biosciences UK Limited, Little Chalfont Buckinghamshire, UK), 28 µl extracted viral RNA and 17 µl RNAse free water was added to an Illustra Ready-To-Go RT-PCR bead (GE-health care, Uppsala, Sweden) and the reaction was performed at 42°C for 60 minutes followed by inactivation of the enzymes at 95°C for 5 minutes.

In order to amplify the NoV capsid P2 domain, a PCR reaction containing 45 µl PCR SuperMix high fidelity (Invitrogen, Carlsbad, USA), 1 µl 10 µM Val fw1 (5′-GAA CTA AAC CAT TCT CTG TCC C-3′) as forward primer, 1 µl 10 µM Val rv1 (5′-AAG TGC TGC ACC CA CTC CTG-3′) as reverse primer, and 2 µl cDNA was mixed. The PCR reaction was performed at 94°C for 5 minutes followed by 35 cycles of 94°C for 30 seconds, 53°C for 30 seconds and 68°C for 1 minute, before a final elongation at 68°C for 10 minutes. PCR-products were visualized on a 1% agarose gel, using EtBr staining and UV light. The final PCR amplicon had a length of ∼700 bp (nt667 to nt1382).

### Sequence analysis

Nucleotide sequencing of the P2 region was performed by Macrogen Inc. (Seoul, South Korea). The sequencing reaction was based on BigDye chemistry, using forward primer Val fw1 and reverse primer Val rv1 as sequencing primers. The amplicons were sequenced twice in each direction, and complete sequences were obtained by assembling overlapping contigs with DNASTAR (DNASTAR, Inc., Madison, Wisconsin, USA). Multiple sequence alignment of NoV capsid proteins was performed, using the ClustalW 1.8 algorithm with default parameters on the European Bioinformatics Institute server. This data was also used for constructing a phylogenetic tree of the outbreak strain capsid P2 domain and reference strains. The phylogenetic tree was constructed using the UPGMA clustering method with distance calculation using the Poisson correction for evolutionary rate by Molecular Evolutionaty Genetics Analysis (MEGA version 4.1).

### Statistical analysis

Fisher's exact test (two-sided) was used to test significant differences in distribution of secretor-positive and secretor-negative individuals among symptomatic and asymptomatic/non-exposed. Mann-Whitney test was used to compare ELISA absorbance values between secretor-positive and secretor-negative individuals as well as individuals with different Lewis phenotypes (Le^a+b−^, Le^a−b+^ and Le^a−b−^). SPSS 16 for Mac was used to perform these analyses and a P-value of <0.05 was considered statistically significant.
